# Multidrug-Resistant *Escherichia coli* in Bovine Animals, Europe

**DOI:** 10.3201/eid2209.160140

**Published:** 2016-09

**Authors:** Evan Brennan, Marta Martins, Matthew P. McCusker, Juan Wang, Bruno Martins Alves, Daniel Hurley, Farid El Garch, Frédérique Woehrlé, Christine Miossec, Leisha McGrath, Shabarinath Srikumar, Patrick Wall, Séamus Fanning

**Affiliations:** University College Dublin, Dublin, Ireland (E. Brennan, M. Martins, M.P. McCusker, J. Wang, B. Martins Alves, D. Hurley, L. McGrath, S. Srikumar, P. Wall, S. Fanning);; Northwest A&F University, Yangling, China (J. Wang);; Vétoquinol SA, Lure, France (F. El Garch, F. Woehrlé, C. Miossec)

**Keywords:** multidrug-resistant, MDR, Escherichia coli, colistin resistance, food-producing animals, bovine, Europe, bacteria, bacterial infection, mcr-1 gene, Enterobacteriaceae, cattle, bovids, antimicrobial resistance

## Abstract

Of 150 *Escherichia coli* strains we cultured from specimens taken from cattle in Europe, 3 had elevated MICs against colistin. We assessed all 3 strains for the presence of the plasmid-mediated *mcr-1* gene and identified 1 isolate as *mcr-1*–positive and co-resistant to β-lactam, florfenicol, and fluoroquinolone antimicrobial compounds.

The dissemination of mobile genetic elements containing antimicrobial resistance genes and the emergence of carbapenem β-lactamases (e.g., *Klebsiella pneumoniae* carbapenemase–2 and New Delhi metallo-β-lactamase-1) have narrowed the chemotherapeutic options available to clinicians (*1*,*2*). Treatment of infections associated with carbapenem-resistant *Enterobacteriaceae* requires the use of polymyxin B and polymyxin E (colistin). These cationic peptides are considered to be the last line of defense for infections in humans. 

Colistin is a drug with a bactericidal action that targets the lipid A component of the lipopolysaccharide structure located in the outer wall of some gram-negative bacteria. Consequently, the drug exhibits a broad spectrum of activity against *Enterobacteriaceae* (*3*). Despite its use in animal production in certain countries, rates of resistance to colistin have so far remained low in animals and humans (*3*,*4*). Polymyxin resistance can develop after modification of the lipid A component in the lipopolysaccharide structure through mechanisms that are chromosomally mediated and result in a reduction in the affinity for these cationic peptides (*5*,*6*). In a recent report, Liu et al. (*7*) described the first known case of plasmid-mediated colistin resistance involving the *mcr-1* gene coding for a phospoethanolamine transferase-like enzyme.

Considering the importance of colistin in the control of multidrug-resistant (MDR) nosocomial human infections caused by gram-negative bacteria and the use of this drug in veterinary medicine, the identification of the *mcr-1* gene in food-producing animals is of major public health importance. The objective of our study was to retrospectively investigate a large collection of *E. coli* cultured from cattle that had suspected enteric or mastitic infections.

## The Study

During 2004–2010, we cultured 150 *E. coli* strains from fecal samples collected from cattle with suspected enteric infection or milk-aliquots collected from cattle with suspected mastitis in France and Germany. We conducted antimicrobial susceptibility testing by using disk diffusion against a panel of 17 compounds consisting of penicillin G, amoxicillin, and amoxicillin/clavulanic acid; cephalothin, cefoxitin, cefotaxime, and cefepime; ertapenem, meropenem, and imipenem; marbofloxacin, ciprofloxacin, and nalidixic acid; gentamicin; tetracycline; florfenicol; and trimethoprim/sulfamethoxazole. We interpreted results according to the criteria of the Clinical and Laboratory Standards Institute where appropriate (*8*,*9*).

A subset of these *E. coli* (n = 45) were classified as MDR and expressed resistance to >3 drug classes. We determined plasmid profiles and PCR-based replicon types as described previously (*10*,*11*) and detected plasmids ranging in size from 2 to 200 kbp. Our PCR-based replicon type analysis identified several incompatibility (Inc) types, including IncX4 in *E. coli* strain 11-1896 and the previously reported IncHI2 type in *E. coli* strain 29957 (Table). We then determined the MICs of these 45 MDR isolates for colistin by using broth microdilution. Three of 45 demonstrated MICs >2 mg/L, which we interpreted as being colistin resistant based on breakpoint tables of the European Committee on Antimicrobial Susceptibility Testing (*12*). We identified these isolates as *E. coli* 22134 O9:H9 U/ST10, *E. coli* 11-1896 O9:H12 U/ST58, and *E. coli* 29957 O101:H9 A or C/ST167 (Table). All were additionally resistant to >2 drug classes, including aminoglycosides, aminopenicillins, cephalosporins, fluoroquinolones, phenicols, tetracyclines, and trimethoprim and sulfonamides. One of the 3 isolates (*E. coli* 29957) was resistant to all of the antimicrobial compounds tested, including β-lactams, florfenicol, and fluoroquinolone compounds (Table). In addition, PCR results indicated that this isolate was positive for the presence of the *mcr-1* gene (Technical Appendix Figure, panel A) (*7*).

**Table:** Selected characteristics of 3 colistin-resistant *Escherichia coli* isolates cultured from cattle with suspected enteric or mastitic infections, France and Germany, 2004–2010*

*E. coli* isolate	Year of isolation	Phylotype	ST	Plasmid size, kbp	PBRT†	Antimicrobial resistance profile	Antimicrobial resistance genotypes†	Colisitin MIC, mg/L
22134	2004	U	ST10	147; 57; 36	IncFIB, IncFIC, IncFII	AML, NAL, CT	*bla*_TEM-1B_, *strAB, tet*(34), ***gyrA, parE, pmrA, pmrB***	8
11-1896	2010	U	ST58	147; 120; 36; 28; 22; 15; 2	IncFIB, IncFII, IncI1, IncQ1, IncX4	AML, CT, CTX, KF, TE, STX	*bla*_CTX-M-1_, *bla*_TEM-1B_, *strAB, sul2, tet*(A), *tet*(34), *dfrA5,* ***gyrB, pmrA, pmrB, phoB, eptB***	8
29957	2007	A or C	ST167	200; 147; 36	**IncFIA, IncFIB,** IncFIC, IncFII, **IncH12,** IncHI2A, IncQ1	AMC, AML, CN, CIP, CT, FLO, MAR, NAL, TE, STX	*bla*_TEM-1A_*, bla*_TEM-1B_*, aadA1, aadA2, aadB, aph*(3′)*-Ia, aac*(3)*-Iia, strAB, sul1, sul2, sul3, tet*(A),* tet*(B)*, tet*(34), *dfrA1, mcr-1, mef*(B), *catA1, cmlA1, floR, ****gyrA, parC, pmrB***	4
*With the exception of *mcr-1* in *E. coli* 29957, genes shown in bold are located on the chromosome in which nonsynonymous amino acid substitutions were identified in the corresponding proteins (online Technical Appendix Tables 1, 2, http://wwwnc.cdc.gov/EID/article/22/9/16-0140-Techapp1.pdf) known to confer resistance to quinolones and colistin. Plasmid replicon types and *mcr-1* genes shown in bold were further confirmed by PCR. AMC, amoxicillin/clavulanate; AML, amoxicillin; CN, gentamicin; CT, colistin; CIP, ciprofloxacin; CTX, cefotaxime; FLO, florfenicol; Inc, incompatibility type, KF, cephalothin; NAL, nalidixic acid; MAR, marbofloxacin; PBRT, PCR-based replicon types; TE, tetracycline; ST, sequence type; STX, trimethoprim/ sulfamethoxazole. †Indicates plasmid replicon types and antimicrobial resistance genotypes extracted from whole genome sequencing data.								

We conducted whole-genome sequencing of 3 isolates with increased MICs for colistin by using the Nextera XT DNA Library Preparation Kit and the Illumina MiSeq platform (Illumina, Inc., San Diego, CA, USA) to produce 300-bp paired end reads (v3 chemistry). We assembled these data de novo using SPAdes version 3.6.2 (http://bioinf.spbau.ru/spades) and then generated queries by using the PlasmidFinder 1.3 (https://cge.cbs.dtu.dk/services/plasmidfinder) and ResFinder 2.1 (http://cge.cbs.dtu.dk/services/resfinder) databases to identify plasmid replicon types and antibiotic resistance genes using BLAST+ (https://blast.ncbi.nlm.nih.gov/Blast.cgi). Several antibiotic resistant genotypes, including some that were acquired, were identified. Four of these genotypes occurred in *E. coli* 22134 isolates, 8 in *E. coli* 11-1986 isolates, and 21 in *E. coli* 22957 isolates (Table). These isolates harbored genes or mutations that confirmed the phenotypes detected in most of the suspected cases of infection in the cattle in our study. We also identified plasmid replicons in all 3 isolates, including 3 types in *E. coli* 22134; 5 in *E. coli* 11-1986, and 7 in *E. coli* 29957. We did not detect the *mcr-1* gene in *E. coli* 22134 or *E. coli* 11-1896; however, we identified several nonsynonymous amino acid substitutions in genes previously shown to be associated with colistin resistance, including *pmrA* and *pmrB*. We also identified *phoP* and *eptB* in *E. coli* 11-1986. Similarly, we identified the *mcr-1* gene in *E. coli* 29957 (a feature that was previously confirmed by PCR) and 1 nonsynonymous substitution in *pmrB*. The *mcr-1* gene was located in a 4,752-bp contig, which when used to query the current databases matched an identical region containing a transposase gene, a phosphoethanolamine transferase gene (the *mcr-1* encoding gene), a hypothetical protein/phosphoesterase gene, and another transposase. The *mcr-1* gene was 100% similar at the nucleotide level to that reported in China and was found to be located distal to the same insertion sequence element IS*Apl1* that mapped to the IncHI2 type plasmid pHNSHP45 (Technical Appendix Figure, panel B) (*7*).

## Conclusions

Plasmid-mediated colistin resistance identified in MDR bacteria of animal origin represents a serious risk to public health. Our data further support recent findings demonstrating that the *mcr-1* gene is not just present in Asia but can also be found in some countries in Europe (e.g., the *mcr-1* gene identified in an *E. coli* strain cultured from a food-producing animal in France in 2007) (Table). Other arrangements of the *mcr-1* gene on plasmids can occur, such as that observed in the IncX4 type (*13*). Liu et al. (*7*) reported that plasmid pHNSHP45 exhibited an in vivo transfer rate between different *E. coli* strains (measured at 10^−1^ to 10^−3^ per recipient) (*7*), a feature that could contribute to the successful dissemination of the *mcr-1* gene. Similarly, in our study, we can also confirm the transfer of the *mcr-1* gene from *E. coli* 29957 via conjugation, albeit at a reduced frequency (data not shown). Especially concerning is the extensive resistance profile of *E. coli* 29957, a feature noted in other studies, which have indicated that colistin resistance might be co-selected after the use of cephalosporins and other compounds (*14*,*15*).

The *mcr-1* gene has now been reported in food-producing animals and in humans located in different geographic regions. In several of these regions, the gene was linked to extended-spectrum β-lactam and florfenicol resistance in the same bacterial isolate (*15*). Because *E. coli* 29957 was identified in 2007, this finding cannot be considered a recent occurrence. Given the genetic mapping reported to date, selective pressure imposed after the administration of broad-spectrum cephalosporins and other compounds might have the potential to co-select for colistin resistance and vice versa, thereby contributing to the dissemination of *mcr-1* (*15*). Molecular epidemiologic studies are required to discover the origin and means of transmission of this gene as a first step in attempting to limit its dissemination, particularly among pathogenic bacteria that threaten human health.

Technical Appendix An agarose gel showing the results of a PCR assay designed to detect the *mcr-1* resistance encoding gene. Nonsynonymous amino acid substitutions identified in genes associated with resistance to nalidixic acid, fluoroquinolones, and colistin.
