# Supplementary material for: Multidrug-Resistant Escherichia coli in Bovine Animals, Europe
Source: Emerg Infect Dis. 2016 Sep;22(9):1650–2. doi: 10.3201/eid2209.160140 (PMC4994333; doi:10.3201/eid2209.160140)
Supplement: Technical Appendix — An agarose gel showing the results of a PCR assay designed to detect the mcr-1 resistance encoding gene. Nonsynonymous amino acid substitutions identified in genes associated with resistance to nalidixic acid, fluoroquinolones, and colistin. [file 16-0140-Techapp-s1.pdf]

Article DOI: http://dx.doi.org/10.3201/eid2209.160140

Multidrug-Resistant *Escherichia coli* in Bovine Animals, Europe

Technical Appendix

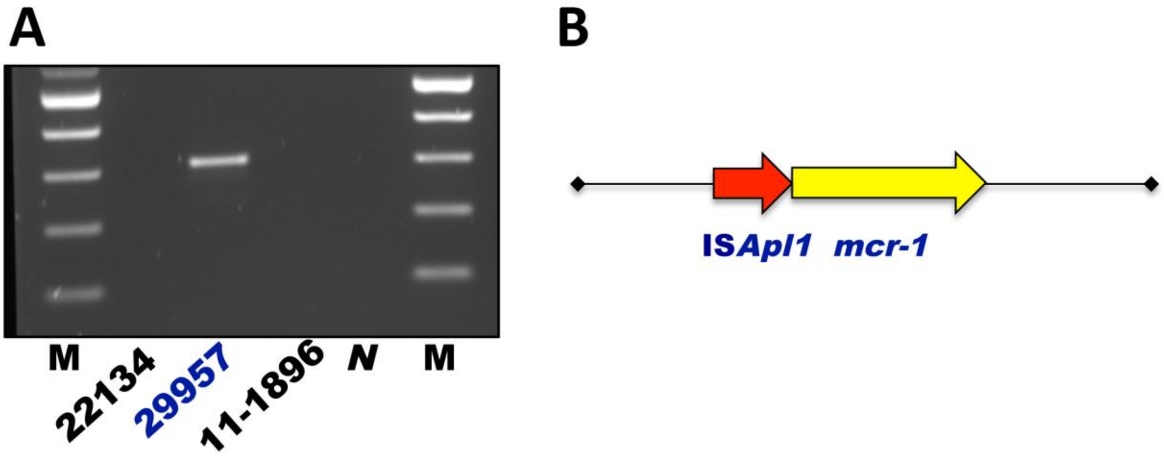

Technical Appendix Figure. PCR assay applied to 3 colistin-resistant *Escherichia coli* isolates cultured from cattle with suspected enteric or mastitic infections, France and Germany, 2004–2014. A) 1% agarose gel showing the *mcr-1* amplicon identified in *Escherichia coli* isolate 29957 (Table). Lane M, molecular weight markers. Lane N, negative control. B) Schematic representation (not to scale) of part of the physical map showing the *mcr-1* gene (at 1,649 bp) located distal to the IS*Apl1* insertion sequence element.

Technical Appendix Table 1. Amino acid substitutions associated with resistance to nalidixic acid and flourorquinolone compounds in 3 *Escherichia coli* isolates cultured from cattle with suspected enteric or mastitic infections, France and Germany, 2004–2010

E. coli isolate	NAL	CIP	MAR	GyrA	GyrB	ParC	ParE
22134	*			S83L			A192V
11–1896					H653R		
29957	*	*	*	S83L D87N		S80I P577L	

*Indicates resistance to this antimicrobial agent as determined by disk diffusion (*1*).

Technical Appendix Table 2. Amino acid substitutions in genes that are associated with resistance to colistin and located on the chromosome in 3 *Escherichia coli* isolates cultured from cattle with suspected enteric or mastitic infections, France and Germany, 2004–2010*

E. coli isolate	*eptB*	*phoP*	*pmrA*	*pmrB*
22134			S29G	D149Y
11–1896	A557T E559V	I44L	S29G G53R	D283G Y358N
29957			S29G	

*Corresponding genotypes were extracted from the whole genome sequence data.

Reference

1. Karczmarczyk M, Martins M, Quinn T, Leonard N, Fanning S. Mechanisms of fluoroquinolone resistance in *Escherichia coli* isolates from food-producing animals. Appl Environ Microbiol. 2011;77:7113–20. PubMed http://dx.doi.org/10.1128/AEM.00600-11
